# Atrial cardiomyopathy in cardiac amyloidosis: clinical imaging and manifestations

**DOI:** 10.1038/s44325-025-00043-z

**Published:** 2025-03-04

**Authors:** Natasha Gorrie, Paul Geenty, Eleanor Rye, Vanathi Sivasubramaniam, Antonia Carroll, Georgia McCaughan, Liza Thomas, Diane Fatkin, Nicole Bart

**Affiliations:** 1 Victor Chang Cardiac Research Institute, Darlinghurst, NSW Australia; 2 Cardiology Department, St Vincent’s Hospital, Darlinghurst, NSW Australia; 3 School of Clinical Medicine, Faculty of Medicine and Health, UNSW, Kensington, NSW Australia; 4 Department of Cardiology, Westmead Hospital, Sydney, NSW Australia; 5 Westmead Clinical School, University of Sydney, Sydney, NSW Australia; 6 St Vincent’s Hospital, Darlinghurst, NSW Australia; 7 SydPath, Darlinghurst, NSW Australia; 8 Faculty of Medicine and Health, Brain and Mind Centre, Sydney University, Camperdown, NSW Australia; 9 Garvan Institute of Medical Research, Darlinghurst, NSW Australia; 10 School of Clinical Medicine, South West Clinical School, University of NSW, Sydney, NSW Australia

**Keywords:** Heart failure, Cardiac hypertrophy, Atrial fibrillation, Cardiovascular genetics

## Abstract

Cardiac amyloidosis is a progressive infiltrative disease and an important cause of atrial arrhythmias, stroke and heart failure. Abnormal amyloid fibril deposition throughout the heart leads to a host of clinical manifestations and complications. Although atrial abnormalities are typically regarded as a consequence of ventricular diastolic dysfunction or atrial arrhythmias, there is emerging evidence that primary defects of atrial structure and function may be present. An atrial cardiomyopathy may be a sign of early cardiac disease, and an unrecognised independent marker of worse prognosis.

This review summarizes current evidence specifically for atrial cardiomyopathy in cardiac amyloidosis, with a focus on imaging and clinical outcomes.

## Introduction

Atrial cardiomyopathy is a loosely defined disorder that is associated with structural, functional and/or electrical remodelling of the atria. It can provide a substrate for atrial arrhythmias, predominantly atrial fibrillation (AF), and increases the risk of thromboembolic stroke, even in the absence of AF. There are numerous causes of atrial cardiomyopathy, the aetiology of which may be genetic or acquired. In this review, we focus on atrial cardiomyopathy in the context of an infiltrative pathology, cardiac amyloidosis (CA). Whilst there is no established definition or clinical criteria for atrial cardiomyopathy in CA, specific features include increased wall thickness, increased chamber stiffness and ultimately chamber dilatation as a late sign of disease. It is also associated with a higher prevalence of atrial arrhythmias compared with other causes.

Historically, research in CA has focused on left ventricular (LV) abnormalities with atrial structural and functional changes presumed to arise consequent to LV dysfunction or AF-induced remodelling. Over the last decade, there has been increasing appreciation that atrial involvement in CA may coincide with, or even precede changes in LV function^1^. Histopathological data suggests that the atria are one of the first sites for amyloid deposition and clinical evidence of this early atrial dysfunction can be seen on imaging, in the absence of atrial arrhythmias or ventricular changes^2,3^. Collectively, these observations highlight the clinical importance of amyloidosis-related atrial cardiomyopathy. There has been an explosion of research recently and here we provide an updated overview of mechanisms, imaging findings, and clinical consequences of this disorder.

### Study search

A PubMed/MEDLINE database search was performed to identify studies from 2005 to 2024 in the English language related to amyloidosis and atrial myopathy. Reference lists of articles were inspected to identify additional papers. Higher quality studies, such as randomized, placebo-controlled, clinical trials and longitudinal observation studies received priority for inclusion. Expert consensus clinical practice guidelines/scientific statements also received priority for inclusion. Given the design of this work as a narrative review, no formal criteria for study selection or appraisal were enforced.

### What is amyloidosis?

Amyloidosis is an umbrella term used to describe a heterogeneous group of conditions characterized by extracellular tissue deposition of misfolded, insoluble, fibrillar proteins, or an amyloidogenic protein. Changes in protein conformation may occur as a result of various processes including abnormal proteolysis and post-translation modifications. Overwhelmed homoeostatic clearance of abnormal protein promotes amyloid fibril formation and deposition in tissues, with subsequent organ dysfunction^4^. Cardiac organ involvement has the highest morbidity and mortality in all subtypes of amyloidosis.

**Fig. 1:**
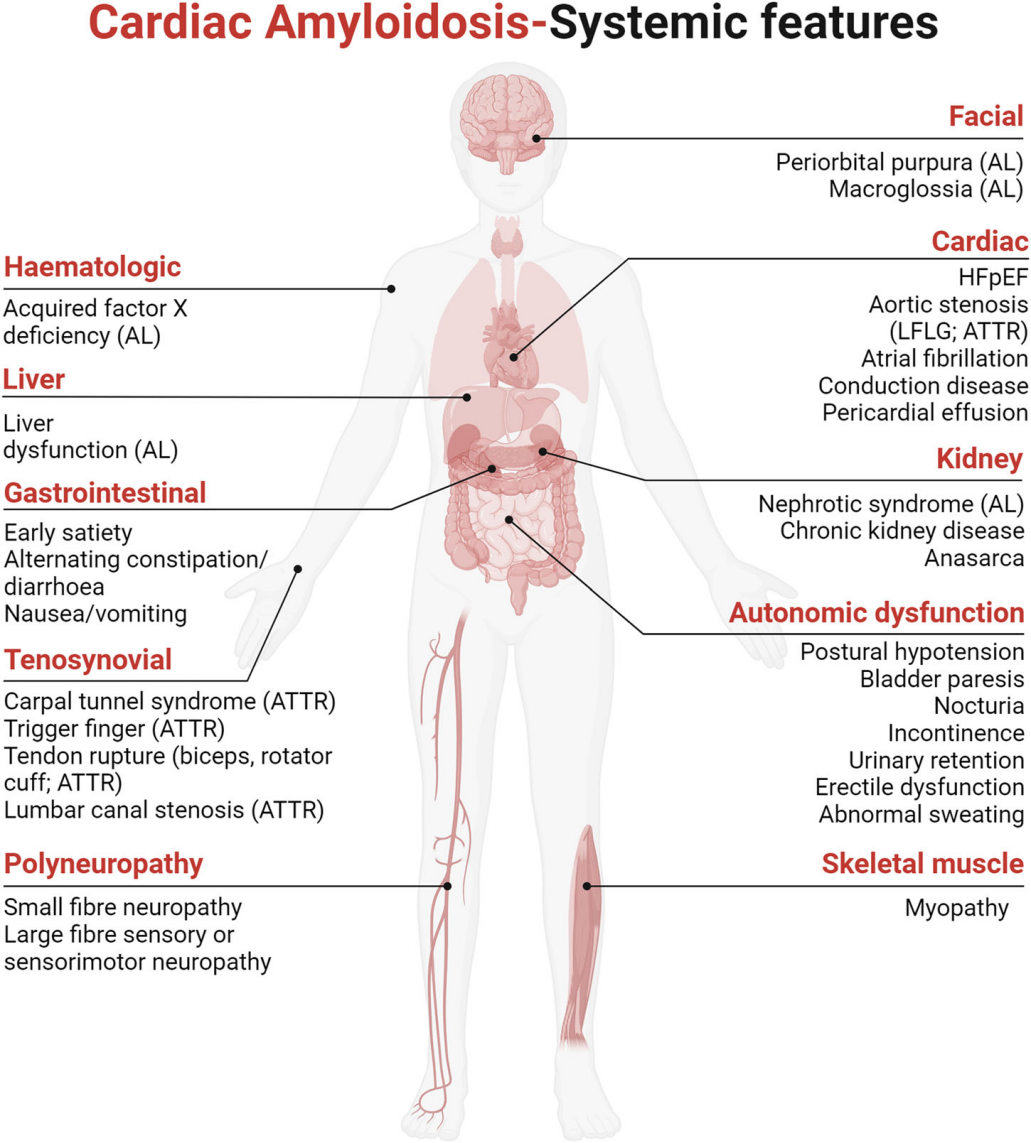
Clinical features in AL and ATTR amyloidosis. AL and ATTR can have numerous clinical features in addition to cardiac involvement, many of which are seen in both subtypes. Features that are distinctive to one subtype (parentheses) are shown. *AL* light chain amyloidosis, *ATTR* transthyretin amyloidosis, *HFpEF* heart failure with preserved ejection fraction, *LFLG* low-flow low-gradient.

Amyloidosis can be localized, with protein generation and deposition at a single site, or systemic, which typically results in extensive disease and multiorgan dysfunction. Over 60 types of amyloidogenic proteins have been identified to date, with more than 35 of these associated with diseases in humans^5,6^. In more than 97% of cases, systemic amyloidosis with cardiac organ involvement is associated with the deposition of immunoglobulin light chains (termed “AL”) or transthyretin (termed “ATTR”). Although these two subtypes of amyloidosis have distinct extra-cardiac clinical features, such features alone cannot reliably distinguish the conditions due to overlap in clinical features (Fig. 1). Isolated, localized atrial CA (termed “IAA” or “AANP” for atrial natriuretic peptide amyloidosis) is also reported but poorly described^7^.

### Types of cardiac amyloidosis

#### Light chain amyloidosis

AL amyloidosis is due to excessive and/or abnormal immunoglobulin kappa or lambda light chain production from clonal or malignant plasma cells. In approximately 50% of cases, AL amyloidosis occurs in isolation, with plasma cell clone bone marrow infiltrates of less than 10%^8^. AL amyloidosis may also occur in association with other plasma cell dyscrasias including multiple myeloma, monoclonal gammopathy of uncertain significance, Waldenström macroglobulinaemia or others.

#### Transthyretin amyloidosis

ATTR amyloidosis results from abnormal protein folding of the transthyretin protein, formally known as pre-albumin. There are two main subtypes of ATTR: (i) Wild-type ATTR (ATTRwt), previously termed 'senile systemic amyloidosis', where normal protein misfolds due to complex and poorly elucidated mechanisms thought to be linked to age-related changes in homoeostatic mechanisms^9^; while (ii) Variant ATTR (ATTRv) results from protein abnormalities that arise due to pathogenic variants in the *TTR* gene, which encodes transthyretin (TTR). TTR is a plasma protein produced predominantly in the liver, with small amounts produced in the choroid plexus and retinal epithelium, that functions as a minor carrier of thyroxine and with 5-retinol binding protein 4 for vitamin A transfer. Normally, TTR exists as a homo-tetrameric structure but if destabilized its tetramers can disassociate into monomers and dimers, forming insoluble amyloidogenic proteins. The *TTR* gene is located on chromosome 18 and is comprised of four exons. More than 130 disease-associated *TTR* variants have been described^10^. *TTR* variants are usually inherited in an autosomal dominant fashion with incomplete penetrance and variable expressivity. Affected individuals may present on a spectrum of predominant neurological, cardiomyopathic or mixed presentation^4^. The predominant cardiomyopathic group typically presents in the 6th or 7th decades.

#### Isolated atrial amyloidosis

IAA is not well understood but is thought to result from overproduction and deposition of the paracrine hormone, atrial natriuretic peptide (ANP). ANP is a natriuretic peptide synthesized and stored in atrial myocytes, which is then released in response to atrial distension from volume expansion. IAA is associated with increasing age and occurs distinct from other forms of amyloidosis, although ANP deposition can co-exist with other forms of CA^11,12^. Histopathologically, ANP deposition is demonstrated predominantly along the sarcolemma of myocytes, with some cases having concurrent brain natriuretic peptide (BNP) deposition^13^. Surprisingly, there is provisional evidence that IAA may have an inverse relationship with fibrosis, although this has yet to be fully elucidated^13,14^. IAA may occur in conditions associated with left atrial (LA) volume overload such as mitral regurgitation but can also be seen in patients with AF or chronic coronary artery disease. Autopsy studies demonstrate IAA in more than 50% of non-amyloid AF cases, with higher rates seen in patients with longer disease duration or with permanent versus paroxysmal AF^7^. However, the underlying mechanism of IAA is more complex than simply atrial stretch and ANP generation, with a surgical series reporting IAA in 39% of atrial tissue specimens from patients with valvular heart disease and persistent AF, but only 7% of those in sinus rhythm with chronic, severe heart failure^15^. IAA has also been reported to occur without structural or electrical myocardial changes^16^. Whilst IAA is known to be associated with a higher risk of atrial arrhythmias, a retrospective study of 167 patients undergoing elective cardiac surgery demonstrated that the presence of IAA was associated with higher rates of peri-operative and one-year mortality, as well as permanent pacemaker implantation^17^.

Loss-of-function variants in the *NPPA* gene, which encodes ANP, are associated with a familial form of early-onset AF^18^. Whilst the disease mechanism is not fully determined, mutant ANP levels have been demonstrated to be significantly higher in affected individuals compared to wild-type ANP in controls^19^. Murine models have demonstrated that mutant ANP results in markedly accelerated oligomer formation with higher intracardiac tissue ANP concentrations and remodelling of sodium, potassium, and calcium channels in mutant mice^18,20^.

#### Other forms

Cardiac involvement is less common in rarer amyloid forms but can occur in apolipoprotein I and IV subtypes and less frequently in serum amyloid A, beta2-microglobulin and others^21^.

### Epidemiology of cardiac amyloidosis

CA is considered a rare disease, although epidemiology is not well elucidated. A multicenter study in Italy reported CA prevalence of 23.5 cases per million, with an annual increase of 17%^22^. The incidence of AL amyloidosis increases with patient age, with the overall burden of both AL and ATTR amyloidosis increasing in our ageing population. The current prevalence of AL amyloidosis is estimated at 5.1 to 12.8 cases per million person-years, with 50–70% having cardiac involvement^23^. There are currently no accurate data for the prevalence of ATTR amyloidosis. Although some amyloidosis centres still report a higher prevalence of AL than ATTR amyloidosis, it is thought that ATTR amyloidosis is likely more common than previously appreciated^24^.

Despite rapid increases in diagnostic rates, it is estimated that a large number of cases remain unrecognized, with a recent multicenter study demonstrating early diagnosis was still very rare^25,26^. Histopathological studies are discrepant, with some series reporting intracardiac TTR amyloid deposition in as many as 1 in 6 autopsy samples, questioning the relationship between histopathological findings and clinical disease. Additionally, a high prevalence of ATTR amyloidosis is reported in the elderly and in specific patient groups, including those with heart failure with preserved ejection fraction (HFpEF), and low-flow low-gradient aortic stenosis^27,28^. A recent multicenter study of routine echocardiographic screening in patients 55 years and older suggested that 29% of patients with hypertrophic, non-dilated LV with preserved ejection fraction and other amyloid red flag features have CA^29^.

The prevalence of ATTRv within ATTR CA cohort also remains poorly identified due to a number of factors including limited and selective clinical and genetic screening. Whilst some data suggest that 5–10% of ATTR CA patients harbour pathogenic *TTR* variants, a recent large study demonstrated that these variants were present in up to 20% of elderly CA patients^30^. Specific *TTR* variants are endemic in some ethnic groups, such as the predominant cardiomyopathic Val122Ile (p.Val142Ile) variant in the African American population, where prevalence is 3–3.5%^31^. Val30Met (p.Val50Met) is the most common variant internationally, endemic in countries such as Portugal, Japan and Sweden. Val30Met carriers variably present with predominant neurologic, cardiomyopathic or mixed phenotypes. Phenotypic expression may be related to fibril length, with one study demonstrating patients with TTR amyloid fibril fragments were more likely to have a late age of onset and cardiac involvement, contrasting to those with only full length fibrils, who had early age of onset and no cardiac involvement^32^.

### Pathophysiology of cardiac amyloidosis

**Fig. 2:**
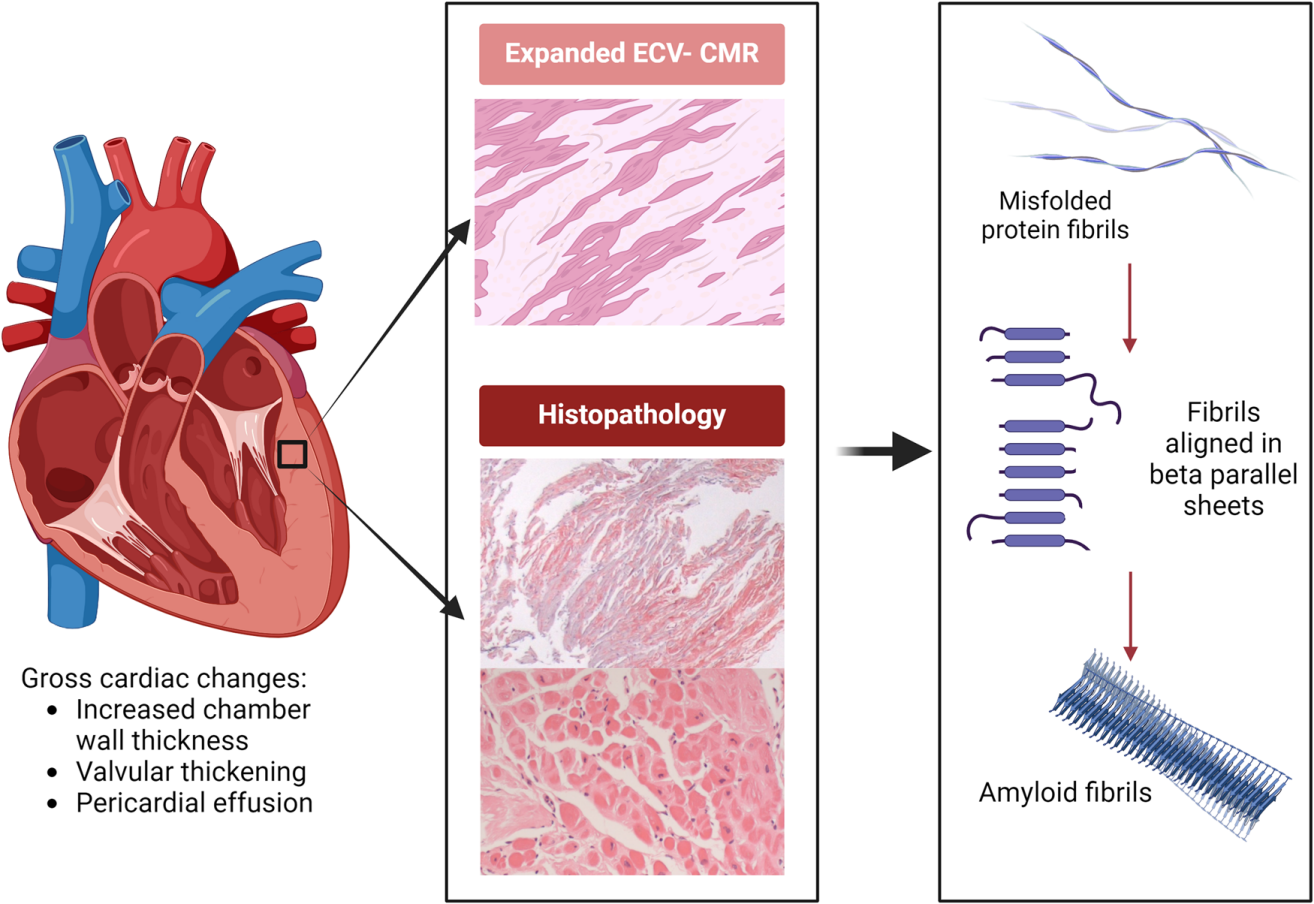
Pathophysiology cardiac amyloidosis (CA). Cardiac changes associated with CA are shown (left panel); increases in left ventricular and right ventricular wall thickness result from interstitial amyloid deposition and expanded extracellular volume (ECV). Expanded ECV is demonstrated with T1 sequences on cardiac magnetic resonance (CMR) imaging (centre panel, top). Histopathology demonstrates Congo red staining under polarised light microscopy (centre panel, middle) with H&E staining showing myocyte attenuation and atrophy (centre panel, bottom). Misfolded proteins align in beta pleated sheets and form insoluble amyloid fibrils (right panel).

Amyloid fibrils form beta-pleated sheets that are aligned in an antiparallel manner and stabilised by intermolecular hydrogen bonds. These are highly resistant to proteolysis and ultimately promote monomer aggregation. This 3D structure is identified histopathologically by Congo red staining, which attaches between the amyloid fibrils, and produces apple-green birefringence under polarised light (Fig. 2). All amyloid fibrils have an affinity for Congo red but alternative stains can be used. Mass spectrometry via proteolysis is gold standard for identification of amyloid subtype but immunohistochemistry and immunoelectron microscopy can also be used with lower sensitivity and specificity^33^.

Deposition of amyloid fibrils is variable in location, distribution and extent. In the heart, deposition predominantly occurs in the subendocardial interstitium, disrupting myocyte architecture and is associated with increased fibrosis. Two patterns of interstitial deposition are recognised; pericellular, with fibrils surrounding cardiomyocytes leading to atrophy, or nodular, which typically alters adjacent myocardial structure^33^. In ATTR amyloidosis, amyloid deposits are typically nodular and diffuse but can be localised or multifocal, whilst in AL amyloidosis the pattern is diffuse, peri-cellular and endomyocardial^4^.

There is often associated vascular deposits, both in intramural and epicardial vessels, which is thought to drive ischaemic symptoms in a significant proportion of patients who do not have obstructive epicardial disease^33,34^. Additionally, coronary microvascular dysfunction is highly prevalent in CA patients, even with no evidence of epicardial disease^35^. In AL amyloidosis, immunoglobulin light chains are postulated to have a direct toxic effect on the myocardium and studies have shown specific organ tropism. Light chains may increase oxidative stress and apoptosis by shifting the cellular redox state^4^. There is also provisional evidence of cellular toxicity in ATTR amyloidosis^4^. One small histopathological study of ATTR amyloidosis demonstrated a lack of acute myocyte injury but highlighted myocyte atrophy, attenuation and thinning^12^. These changes ultimately lead to decreased atrial elasticity, contractility and compliance, which can increase atrial filling pressures.

Whilst limited atrial specific studies have been performed; some case reports demonstrate concurrent ANP deposition with TTR in atrial histopathology^36^. The largest series of detailed atrial assessments reviewed five cardiac explants with ATTR (three wild-type, two variant). All samples demonstrated extensive TTR deposition in the interstitum and subendocardium, three cases were diffuse and two were multifocal. Concurrent ANP was demonstrated in three cases^12^.

There is still much to be learnt regarding the time course and distribution of amyloid fibril deposition. A retrospective autopsy study demonstrated the highest fibril burden in the LV basal segments, with a gradient to the apex, correlating with wall thickness on echocardiography^37^. Basal deposition may be accompanied by microvascular ischaemia with further impairment in function^35^. A recent histological study demonstrated that deposition of amyloid fibrils in the atria occurred concurrently with ventricular deposition, with early predilection for the interatrial septum and the basal ventricular septum^2^. These findings differ from clinical observations in which LV basal septal changes were seen first^38^. It has also been proposed that atrial amyloid deposition precedes ventricular deposition in some cases^3^. A three-patient case series demonstrated histopathological evidence of ATTR CA in atrial and valvular tissue, but no evidence of ventricular change on imaging^39^. Interestingly, unlike clinical prevalence data that describe a male predominance of 7:1, there are no gender differences reported in frequency of amyloid deposition in cardiac tissue analyses, questioning gender differences in CA diagnostics and clinical disease^2,40^.

Whilst histopathology is the gold standard for diagnosis of atrial CA, this traditionally requires surgically obtained atrial tissue samples. Non-surgical atrial sampling techniques remain largely research based and are not widely available. As surgical sampling is not often feasible, in recent years, there has been an explosion of literature on the use of non-invasive cardiac imaging to diagnose atrial cardiomyopathy, with a particular interest in techniques to detect CA.

### Atrial imaging in cardiac amyloidosis

Current imaging techniques largely focus on the role of transthoracic echocardiography (TTE), with fewer studies addressing the role of nuclear imaging, cardiac magnetic resonance imaging (CMR) and computed tomography (CT) (Table 1). Positron emission tomography (PET) is an emerging area of active research. Imaging studies are complicated by high rates of AF and its impact on assessment of parameters of atrial structure and function. There is limited data on the natural history atrial dysfunction over time and imaging modalities to assess these changes^41^. Additionally, data on response to available disease-modifying therapies is limited with regards to atrial involvement.

**Table 1:** Comparison of characteristic and atrial specific findings, strengths and limitations of non-invasive imaging modalities in cardiac amyloidosis

	TTE	CMR	Cardiac scintigraphy	CT	PET
Characteristic amyloid findings	↑LV + RV wall thickness ↑ relative wall thickness ↓ GLS with ‘apical sparing pattern’ Diastolic impairment Atrial dilatation Pericardial effusion	↑LV + RV wall thickness LGE; subendocardial to transmural in LV, also seen in RV + atria Difficulty with myocardial nulling ↑ native T1 signal ↑ extracellular volume %	Increased uptake; visual, semi-quantitative grading, H:CL ratio	Unclear, currently under investigation Increased extracellular volume^79^	Increased myocardial retention index^82^
Specific atrial findings	Dilated atria (diameter, area, volumes)^44^ MV inflow pattern suggestive of advanced diastolic impairment^48^ ↓ LA strain (all phases)^4^	Thickened walls^61^ LGE enhancement^59^ ↑ extracellular volume ↓ LA strain (all phases)^70^	Increased uptake; visual only^73^	-	-
Strengths	Widely available Low relative cost No radiation or contrast	Detailed imaging characteristics Detects early CA changes	Diagnostic in some cases No contraindications	Widely available Low relative cost	Able to identify all amyloid subtypes
Limitations	Cannot differentiate from other cardiomyopathies Cannot differentiate CA subtype Atrial specific windows may be limited (i.e. body habitus) Limited in patients not in SR	Cannot differentiate CA subtype Limited in specific settings (CKD, claustrophobia) Cost and accessibility	Long time of study (>3 h) Radiation exposure Predominantly used for ATTR with low sensitivity for AL+ some variants of ATTRv	Limited to research space currently Radiation exposure	Limited to research space currently Cost

*AL* light chain amyloidosis, *ATTRv* variant transthyretin amyloidosis, *CA* cardiac amyloidosis, *CMR* cardiac magnetic resonance, *CKD* chronic kidney disease, *CT* computed tomography, *ECV* extracellular volume, *GLS* global longitudinal strain, *H:CL* heart to contralateral lung, *LGE* late gadolinium enhancement, *LV* left ventricle, *MV* mitral valve, *RV* right ventricle, *SR* sinus rhythm.

#### Transthoracic echocardiography

TTE studies of CA-associated atrial cardiomyopathy have documented changes in both structural and functional parameters, as well as associations with clinical outcomes.

Methods for estimation of atrial size have evolved over time from the measurement of atrial diameter (M-mode or B-mode images) to LA areas (4-chamber apical view), to LA volumes calculated either from combined biplane 2-dimensional (2D) measures (apical 2- and 4- chamber views), or more recently from real-time 3-dimensional (3D) measures. CA-associated atrial cardiomyopathy has been associated with increased 2D and 3D LA end-diastolic and end-systolic volumes, with similar associations seen with both compared to controls^12,42,43^. LA enlargement was an independent predictor of 5-year survival, even after correcting for LV ejection fraction and septal wall thickness^44^. In AL amyloidosis, LA-indexed volume correlated with Mayo Staging and was an independent predictor of mortality, with larger volumes associated with worse outcomes^45^. LA minimal volume has also been identified as a predictor of mortality of AL CA, albeit in a small cohort^46^. Atrial dilatation occurs in parallel with disease progression and is often a sign of advanced disease^47^.

In many cardiac conditions, changes in atrial function assessed by strain analysis precede volumetric changes^48^. Mitral inflow patterns in CA resemble those seen in other causes of LV diastolic dysfunction e.g. rapid deceleration time of the E wave with marked reductions or absence of the A wave^49^. Historically, LA strain was assessed using tissue Doppler but was largely limited to research studies due to the time required for data acquisition and analysis, and dependency on the angle of the Doppler derived measurement. Speckle tracking imaging has more recently permitted semi-automated assessment of phasic atrial function. Studies in CA have demonstrated impairment in all three phases of atrial function; reservoir, conduit and contractile or ‘booster’ strain, in those in sinus rhythm, independent of LV function^12,42^. Of the three atrial phases, LA reservoir strain has been demonstrated across numerous studies to be most closely associated with clinical outcomes and provides additive value beyond LV parameters prognostically^50^. LA reservoir strain has also demonstrated a role in differentiating CA from other phenocopy conditions^51–53^. Impairments in LA strain parameters were worst in patients with ATTRwt^42^. Reductions in functional parameters correlate with LV dysfunction and with clinical outcomes in CA independent of LV parameters. Together this supports an impairment of both passive and active atrial function in CA^42^.

Studies assessing atrial response to treatment in CA are limited but a recent paper on patients treated with Tafamidis reported improvements in reservoir strain in those in sinus rhythm, but interestingly not in patients with AF^54^.

In patients undergoing stem cell transplantation for AL amyloidosis, those who developed AF had significantly lower LA booster and reservoir strain, with no significant changes in LA volumes. LA reservoir strain has been demonstrated to be more sensitive in diagnosing early AL CA than standard parameters such as LV apical sparing^55^. Additionally, changes in reservoir and contractile LA strain have been demonstrated to predict arterial thromboembolic events and mortality, and were found to be additive to the established CA scores, including both Mayo (AL) and Gillmore (ATTR) staging systems^56,57^. Right atrial (RA) reservoir strain has similarly recently shown additive value to predicting all cause death in AL, in addition to other established measures including LA reservoir strain^57^. RA reservoir strain is also associated with increased mortality in ATTR^58^.

It has been suggested LA strain may have a role in the diagnosis of sub-clinical disease in *TTR* variant carriers. A study demonstrated abnormalities in reservoir and contractile LA strain in Val122Ile variant carriers with sinus rhythm^59^.

Newer parameters including LA stiffness and mechanical dispersion have recently been described in CA. LA stiffness, or resistance to deformation, can also be derived from speckle-tracking imaging. It is measured by the ratio of E/e’ divided by LA reservoir strain and has been demonstrated to be increased in ATTR CA, as well as in *TTR* variant carriers^12,59^. LA mechanical dispersion, a novel marker of intra-atrial dyssynchrony, has been recently demonstrated to be increased in ATTR compared with AL^46^.

Assessment of atrial cardiomyopathy in CA is currently best assessed with echocardiography, however, whilst conduit and reservoir strain may be assessed regardless of rhythm, organised atrial activity is required for assessment of contractile strain, which limits the application of this technique in patients with AF. Additionally, speckle tracking has been unable to be performed in more than a quarter of CA patients in sinus rhythm due to technical factors.

#### Cardiac magnetic resonance imaging

CMR is the gold standard for tissue characterization and volumetric assessment in cardiac disorders. However, CMR studies of CA-associated atrial cardiomyopathy are limited mainly to retrospective cohorts or secondary outcomes. CMR changes seen in the LA mirror those in the LV, including thickened atrial walls, elevated extracellular volume (detected by T1 sequences) and evidence of varying patterns of late gadolinium enhancement (LGE).

Increased extracellular volume is a characteristic feature of CA, as demonstrated in CMR with elevated T1 values. Atrial amyloidosis is associated with increased atrial T1 values, with a meta-analysis demonstrating an associated increase in all-cause mortality and a hazard ratio of 3.95. Extensive atrial LGE has been demonstrated in CA cohorts, disproportionate to other similar cardiomyopathic conditions^60,61^. The amount of atrial LGE correlated with other atrial parameters, including LA active and total emptying fractions, in an AL amyloidosis population.

Increased interatrial wall thickness has been demonstrated in a small study across the CA subtypes, with this being worst in ATTRwt^62^. One study, with a cohort of 14 patients, suggested LA wall thickening was associated with adverse outcomes including pacemaker implantation and the primary composite outcome (all-cause mortality, unplanned cardiovascular hospitalizations, sustained ventricular arrhythmias, permanent reduction in left ventricular ejection fraction, and pacemaker implantation)^63^.

Increases in maximum and minimum LA volumes relative to controls have been shown, even in the absence of changes in LA ejection fraction and emptying volume^64^, but the clinical utility remains an issue. Although atrial volumes increase as the severity of disease increases, bi-atrial enlargement using standard volumetric criteria has been demonstrated to be a late sign of disease progression^65^. Reduction in LA emptying fraction, defined as ([LA maximal-LA minimal volume]/LA maximal volume), has been independently associated with mortality^66,67^. Lower LA emptying fraction in sinus rhythm CA patients has also been demonstrated to be associated with significantly higher rates of incident AF, although both groups were reduced compared to normal populations, again impacting clinical utility^68,69^. Importantly, patients in sinus rhythm with poor LA emptying fraction have demonstrated higher mortality than those with AF^70^.

Studies have demonstrated similar CMR findings to TTE, with reductions in all phases of LA strain in small CA cohorts^71,72^. Whilst there were no differences between AL and ATTR patients in LA geometry in one study, LA function appeared worse in the ATTR cohort^71^. One study demonstrated worse LA reservoir strain, as compared to hypertrophic cardiomyopathy patients, but notably more than 85% of the cohort were NYHA class III or more^72^.

#### Cardiac scintigraphy

Cardiac bone scintigraphy has become the cornerstone for non-invasive diagnosis of CA, specifically ATTR, but there have been few descriptions of atrial features^73^. Atrial uptake on bone scintigraphy has been associated with both significantly higher rates of AF and correlated with histopathological diagnosis of CA but there is no standardised grading or assessment criteria for atrial involvement^74^. Positive scintigraphy scans have previously been thought to result from binding of isotope to micro-calcifications within tissue. Whilst microcalcifications have been demonstrated histopathologically in cardiac tissue from patients with CA (including both ATTRwt and ATTRv), several cases with negative cardiac scintigraphy, particularly some Phe64Leu and Val30Met *TTR* variants, remain unexplained^75^. Additionally, a PET tracer targeting microcalcification has demonstrated limited value in CA detection^76^. One proposed explanation for inconsistent scintigraphy uptake is variation in amyloid fibril type^76^. Type A fibrils, almost always seen in ATTRwt, have both full-length and C terminal containing fibril fragments, which contrasts with type B fibrils, which are only full-length fibrils and are more common in ATTRv.

LA echocardiographic parameters have been correlated with cardiac scintigraphy using techneiumm-99m-3,3-diphosphono-1,2-propanodicarboxylic acid (DPD) in a small study^77^. LA minimum and maximum volume, emptying fraction and reservoir stain demonstrated reasonable correlation to DPD tracer uptake in 40 ATTR patients with receiver operator curves (ROC) demonstrating an area under the curve of >0.8 for all characteristics. Interestingly, one retrospective study of technetium-99m-pyrophosphate (PYP) scintigraphy reported atrial uptake in 20% of the cohort but noted only half of these patients met clinical criteria for the diagnosis of ATTR amyloidosis, which relies on ventricular uptake^3^. Those with atrial uptake but not meeting diagnosis for ATTR were more likely to be women. Whilst not all patients underwent histopathological confirmation, amyloid deposition was demonstrated histopathologically in 13 patients without ventricular uptake, suggesting this may be an avenue for early diagnosis^38^.

#### Computed tomography

Cardiac CT has not traditionally been utilised in CA, with no role to date in the diagnosis or monitoring of disease. Recent work has focussed on extracellular volume quantification and a role in screening for amyloidosis in specific subset populations, similar to TTE^78,79^. One study demonstrated CT-derived extracellular volume fraction in the LV to be predictive of mortality in CA patients^80^. Another study assessed the use of 4-dimensional CT to identify ATTR CA in severe AS patients utilizing LA strain, LV strain, relative apical strain and mass index. Compared with scintigraphy, the study demonstrated a sensitivity and specificity of 96% and 59% respectively if two parameters were abnormal^81^.

CT in other atrial cardiomyopathies has a demonstrated role in prediction of response to ablative therapy and predictive risk of strokes but CT studies of atrial involvement in CA or outcomes have not been reported^82^.

#### Positron emission tomography

PET is an emerging area of interest in CA with current investigational tracers able to bind to amyloid independent of the precursor protein. Two tracers of interest, 18F-florbetapir and 124I-evuzamitide, commonly known as AT-01, appear to bind along the beta-sheet surface via cross-strand ladders or via the abundantly present glycosaminoglycans respectively^76^. These tracers hold promise in Phase 1 and 2 studies with quantification and evidence of uptake with high sensitivity in AL, ATTR and rarer amyloid subtypes^83^. Whilst these tracers likely have a role in future amyloid diagnostics, several limitations exist, including a lack of standardised imaging protocols and prohibitive cost for their widespread use. Further clinical trials, including unselected real-world cohorts are required. There is currently no published work in atrial cardiomyopathy utilising these markers.

### Biomarkers

Whilst biomarkers remain the cornerstone of disease staging and prognostication in both AL and ATTR amyloidosis, there has been little work in CA-associated atrial cardiomyopathy. In CA, as in other cardiomyopathies, cardiac biomarkers such as troponin and NT-proBNP, may be elevated in patients in AF and so higher reference ranges are established^84,85^. In this subgroup, biomarkers remain valid for disease prognostication but have decreased performance^86,87^. This is reflected by higher biomarker cutoff values for clinical trial entry in CA. Serum ANP is not used in routine clinical practice but has demonstrated a role as a marker for early acute heart failure^88^. It has no established role in the diagnosis, prognostication or monitoring of CA. Several disease-specific biomarkers have been proposed for use in ATTR amyloidosis, but these have not been researched specifically in the context of atrial cardiomyopathy^89^. Serum TTR concentration has been demonstrated to be reduced in those with clinical disease, with lower levels associated with worse clinical status and survival^87^. Serum TTR levels have demonstrated utility in the assessment of response to disease-modifying therapy and are utilized as an endpoint in some clinical trials.

Specifically in AL amyloidosis, free light chains hold additional prognostic significance with suppression of the abnormal cell clone associated with improved outcomes^89^. However, end-organ progression can occur in the absence of a detectable abnormal immunoglobulin, and there is no definitive research with respect to atrial cardiomyopathy.

### Clinical outcomes in cardiac amyloidosis-associated atrial cardiomyopathy

**Fig. 3:**
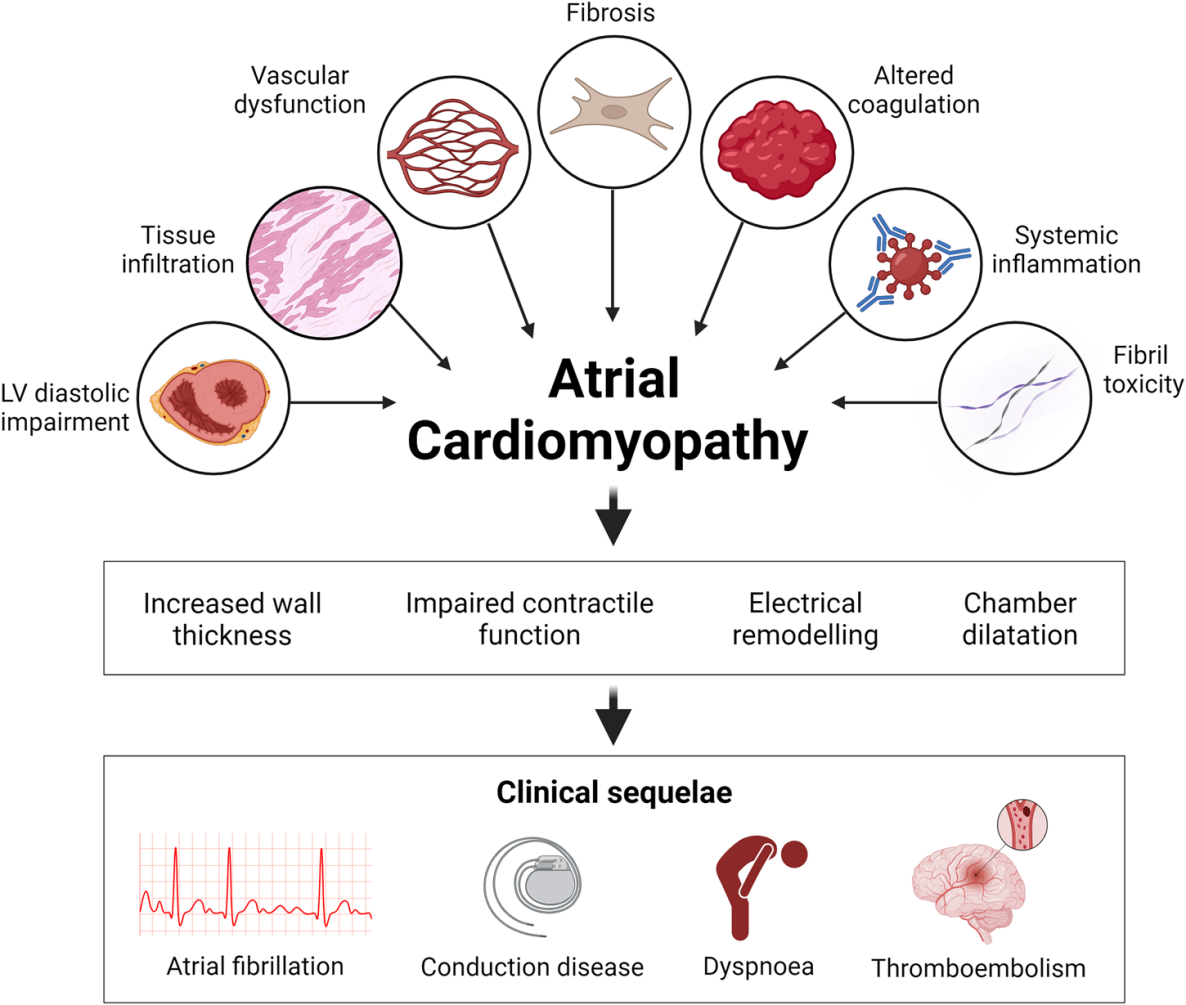
Pathophysiological factors that contribute to atrial cardiomyopathy and clinical outcomes in cardiac amyloidosis. Multiple local and systemic factors, top panel, contribute to atrial cardiomyopathy in CA phenotype, middle panel, ultimately resulting in clinical outcomes, seen in the lower panel.

The clinical consequences of CA-associated atrial cardiomyopathy are similar to those of other cardiomyopathies, with impaired hemodynamics and increased rates of atrial arrhythmias and intracardiac thrombi (Fig. 3).

#### Atrial fibrillation

While AF can result in structural and electrical remodelling of the atria, atrial cardiomyopathy per se can also act as a primary substrate for AF. AF is associated with older age, higher NYHA functional class, LV size and mitral regurgitation. It is also associated with a higher ATTR stage (Gillmore) in several studies. In a small study, AF prevalence was associated with lower AL staging (Mayo 2004), which may reflect the small cohort of AL relative to ATTR in this study^90^.

The prevalence of AF is higher in CA than in any other cardiomyopathies. At the time of CA diagnosis, 30–70% patients have known AF, with prevalence rising to nearly 100% in patients with end-stage ATTR amyloidosis^91^. AF is associated with a 2.5-fold increased risk of stroke in CA compared to patients without CA, and when AF-related strokes occur they are 2.5 times more likely to be fatal^92,93^. Whilst AF rates are heterogeneous across studies, those with ATTRwt have the highest rates at about 40–71%, with ATTRwt the most likely diagnosis when a patient’s first clinical presentation is AF^2,94,95^. Rates also remain high in AL and ATTRv CA, although for ATTRv these vary with the genotype^96^. AF in CA is associated with higher rates of stroke and heart failure but not increased mortality. Analysis of the ATTR-ACT cohort demonstrated AF was an independent prognostic factor for mortality on prespecified covariates but when expanded covariates were used it was not^97^. Additionally, rhythm control does not lower stroke risk in this cohort, supporting the hypothesis that rhythm changes are a manifestation of an underlying atrial cardiomyopathy^92^. A recent study demonstrated absent atrial contraction in almost a quarter of ATTR patients in sinus rhythm, a condition described as ‘atrial electromechanical disassociation’. In that study, there were no significant differences in clinical outcomes between patients with atrial arrhythmias and those with ‘electromechanical disassociation’, which may explain why AF has not been associated with higher mortality in CA^12^.

Despite the significant association with AF, one study assessing CA prevalence in patients with AF with LV hypertrophy over the age of 60 years old demonstrated only 1.3% of the cohort met diagnostic criteria^98^.

Other clinical manifestations may precede AF including bradycardia, pauses and atrioventricular block, although these occur less frequently. CA patients with incomplete or advanced interatrial block have significantly higher rates of AF, underscoring the association with an underlying atrial cardiomyopathy^68^. In ATTR, pacemaker implantation rates are between 10% and 40%^91^. Additionally, AF is independently associated with higher device implantation in CA patients, with 3.8 times higher rate of device implantation at three years follow up in one study^99^.

#### Thromboembolic events

Atrial cardiomyopathy is associated with increased intracardiac thrombus formation and increased arterial thromboembolic events, specifically stroke. The heightened thrombotic risk in CA is not well elucidated but thought to be due to a range of factors including restrictive filling, fibril deposition and impact of LV hemodynamics ultimately leading to LA remodelling. Whilst changes in coagulability are well established in AL CA, thrombus formation in ATTR CA is also significantly higher. This remains incompletely understood but it is posited that TTR may play a role in activation and regulation of the coagulation and fibrinolytic systems^100^.

Intracardiac thrombi are demonstrated in up to one-third of CA patients, being highest in the ATTRwt population, with clinical scores, such as CHADS-VASc and CHA2DS2VASc, being poor predictors of thrombus risk^101,102^. Regardless of subtype, anticoagulation status, including chronic anticoagulation, or cardiac rhythm, studies have consistently demonstrated high rates of intracardiac thrombi on transesophageal echocardiography, CMR and autopsy studies. This is reflected in international guidelines, which recommend transoesophageal assessment for intracardiac thrombi prior to cardioversion in CA patients^5^. Strokes secondary to AF are usually related to LA appendage thrombus but may occur in other LA locations.

Studies have also shown higher thromboembolic event rates in CA compared with the general population, with a recent multicenter study demonstrating an incidence of 2.2% per year, despite a third of this cohort documented in sinus rhythm with no prior history of AF^103^. Another recent study demonstrated 75% of thrombotic events in a CA population occurred without evidence of a prior AF episode. The study also showed those in sinus rhythm at baseline that later develop AF appear to be at the highest risk of thromboembolic events, highlighting the need for clinical tools to identify these patients^56,102^ Despite CA patients being at higher risk of bleeding complications, in the absence of robust predictive algorithms for thromboembolism risk, anticoagulation is a guideline recommendation for those with any evidence of atrial arrhythmias^5,21^. CHA2DS2-VASc may identify those at risk of arterial thromboembolic events if in sinus rhythm in the amyloidosis population^103^, however, these findings have not been borne out in one large international registry^102^. The study demonstrated no thromboembolic events in sinus rhythm patients who were anticoagulated over a median follow-up of 20 months, as compared with incidence rate of 1.3 per 100 patient years in those without anticoagulation^102^. International guidelines have also recommended consideration of anticoagulation for CA patients in sinus rhythm^104^.

### Future directions

Increasingly it is recognised that CA is as much a disease of the atria as the ventricular myocardium; with impaired atrial function, dilatation and electrical abnormalities, although these mechanisms are not fully identified. Non-invasive imaging techniques, particularly echocardiography, have identified changes in both atrial size and function associated with CA. The most sensitive measures appear to be changes in atrial strain, particularly of reservoir and contractile function, which portend worse clinical outcomes.

Several clinical questions remain; what changes in atria are seen throughout the disease in CA? Is there a possible alternate non-invasive diagnostic CA pathway utilizing atrial parameters? What is the response of atrial changes to disease-modifying therapies? Could atrial changes be indications for early use of disease-modifying therapies? Additionally, how do we identify patients with CA who may require anticoagulation but have no history of AF? Can we better risk stratify CA patients with AF when considering anticoagulation? To address these questions research areas must be addressed. Firstly, the imaging characteristics of atrial cardiomyopathy in CA must be defined, particularly on CMR and cardiac scintigraphy, to allow for larger-scale clinical trials and standardised assessment across centres. Secondly, imaging modalities must be compared and analysed to identify the optimal modality for both early diagnosis and disease monitoring. Then longer-term multicenter studies linking atrial cardiomyopathy to clinically relevant outcomes are required. This information would ultimately inform how to best identify patients, particularly those in sinus rhythm, who are at the highest risk for thromboembolic disease. Finally, a randomized clinical trial of anticoagulation in sinus rhythm patients based on a scoring system that incorporates imaging parameters would be required to assess the benefit and safety of this strategy.

## Data Availability

No datasets were generated or analysed during the current study.
